# Functional non-coding polymorphism in an *EPHA2* promoter PAX2 binding site modifies expression and alters the MAPK and AKT pathways

**DOI:** 10.1038/s41598-017-10117-3

**Published:** 2017-08-30

**Authors:** Xiaoyin Ma, Zhiwei Ma, Xiaodong Jiao, J. Fielding Hejtmancik

**Affiliations:** 1 Laboratory of Developmental Cell Biology and Disease, School of Ophthalmology and Optometry and Eye Hospital, Wenzhou Medical University, Wenzhou, 325003 China; 2 Ophthalmic Genetics and Visual Function Branch, National Eye Institute, National Institutes of Health, Bethesda, MD 20892 USA

## Abstract

To identify possible genetic variants influencing expression of *EPHA2* (Ephrin-receptor Type-A2), a tyrosine kinase receptor that has been shown to be important for lens development and to contribute to both congenital and age related cataract when mutated, the extended promoter region of *EPHA2* was screened for variants. SNP rs6603883 lies in a PAX2 binding site in the *EPHA2* promoter region. The C (minor) allele decreased *EPHA2* transcriptional activity relative to the T allele by reducing the binding affinity of PAX2. Knockdown of *PAX2* in human lens epithelial (HLE) cells decreased endogenous expression of *EPHA2*. Whole RNA sequencing showed that extracellular matrix (ECM), MAPK-AKT signaling pathways and cytoskeleton related genes were dysregulated in *EPHA2* knockdown HLE cells. Taken together, these results indicate a functional non-coding SNP in *EPHA2* promoter affects PAX2 binding and reduces EPHA2 expression. They further suggest that decreasing *EPHA2* levels alters MAPK, AKT signaling pathways and ECM and cytoskeletal genes in lens cells that could contribute to cataract. These results demonstrate a direct role for PAX2 in EPHA2 expression and help delineate the role of EPHA2 in development and homeostasis required for lens transparency.

## Introduction

Cataract is an opacity of the crystalline lens^1^. Hereditary cataract can occur at or near birth, usually as a Mendelian trait, or as individual ages, as a multifactorial trait influenced by multiple genes and environmental factors. There is increasing epidemiological evidence that genetic factors are important in the pathogenesis of age-related cataract^2^, often through single nucleotide polymorphisms (SNPs) in genes. In some cases, genes implicated in congenital cataracts also have been associated with inherited cataracts having later onset or progression throughout life, suggesting that mutations that completely disrupt the protein or functionality might cause congenital cataracts with highly penetrant Mendelian inheritance, while mutations that cause milder damage might contribute to age-related or progressive cataracts showing reduced penetrance or a multifactorial inheritance pattern. Examples of this include the ‘Osaka’ variant of GALK1 (p.A198V)^3^, CRYAA (p.(F71L))^4^, and in the 5’UTR of SLC16A12 (c.-17A > G)^5^. In addition, several SNPs have been reported to be associated with ARC^6–8^, although the exact mechanisms of cataract initiation have not been identified. One possible mechanism for associations of SNPs that cause no sequence changes in the protein sequence might be alterations in the level of expression of the expressed protein.

Eph-ephrin signaling is essential for lens transparency, and mutations in *EPHA2* (MIM 176946) have been reported to cause human congenital cataracts^9–11^. Additionally, polymorphisms in *EPHA2* also have been linked to ARC in humans^9, 12, 13^. *EPHA2* is highly expressed in the mouse lens, and loss of *EphA2* disrupts the structure and organization of lens fiber cells through altered N-cadherin adhesion junctions^14–16^, causing age-related cortical cataract^14, 15^. Overexpression of EPHA2, promotes the cytoprotective and anti-oxidative capacity of lens epithelial cells, and this protection is lost when the EPHA2 being expressed contains mutations associated with cataract^17^. Mouse lenses in which *Ephrin-A5*, a ligand of EPHA2, are knocked out also displayed disruption of lens fiber cell packing and cataract^18^. These results clearly show that *EPHA2* plays critical roles in lens transparency, although the precise mechanisms have not been determined.

*PAX2* (MIM 167409) is a transcription factor belonging to the PAX (paired box) family. It is co-expressed with PAX6 (paired box 6) in the optic vesicle at around E12.5 mouse, but is highly expressed in the optic nerve at later developmental stages^19^. *Pax2* is also expressed in the retina, otic vesicle, semicircular canals, spinal cord, adrenal glands, and kidney^20^. Functionally, *PAX2* induces *WT1* expression in the mesenchymal transition to epithelium during renal development and is active in repression of *ERBB2* transcription by the estrogen receptor^21^. Mutations in *PAX2* have been implicated in retinal colobomas, including the papillorenal syndrome (PAPRS, MIM120330). *D-PAX2* has been implicated in Crystallin expression in Drosophila^22, 23^. However, while PAX6 has been shown to play a critical role in lens development and cataractogenesis^24, 25^, the functional role of PAX2 in the lens remains largely unknown.

Some non-coding SNPs in a gene’s promoter or enhancer region play critical roles in regulating transcriptional activity^26–28^. We have previously reported that the non-coding SNP rs7278468 is associated with ARC through decreasing transcriptional activity of the CRYAA promoter^29^. In this study, we show that rs6603883 in the promoter region of *EPHA2* is located in a binding motif of PAX2 (paired box 2), and the minor allele decreases PAX2 binding reducing the transcriptional activity of *EPHA2*. Knockdown of *PAX2* in HLE cells decreased expression of both *EPAH2* mRNA and protein. RNA sequencing identified differential expression of 33 genes, including genes in cytoskeleton organization, MAPK and/or AKT signaling pathways, and the ECM, cell membrane, cell surface, or basement membrane. These results suggest that EPHA2 may act in HLE cells through ECM regulation of MAPK and AKT signaling pathways to affect cell cytoskeletal organization and induce cataract formation.

## Results

### rs6603883 lies in the *EPHA2* promoter region and influences the transcriptional activity of *EPAH2*

**Figure 1:**
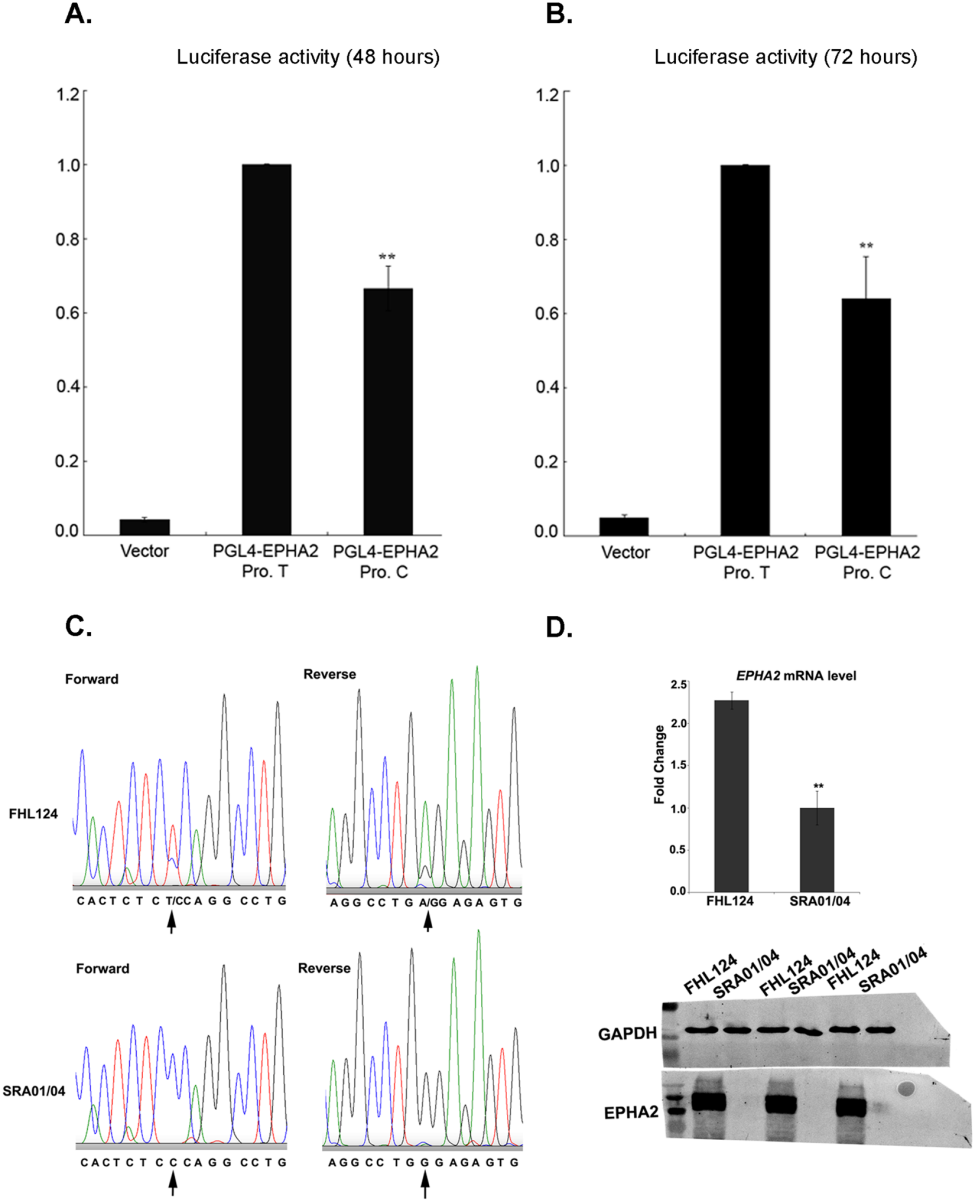
rs6603883 allele specifically regulates the transcriptional activity of *EPHA2*. Luciferase reporter assay to test *EPHA2* transcriptional activity was carried out in HLE cells at 48 (**A**) and 72 (**B**) hours after transfection. The rs6603883 C_C genotype decreased *EPHA2* promoter transcriptional activity significantly. Firefly luciferase activity was normalized to renilla luciferase activity. Error bars represent standard deviations of 3 three independent experiments. **Indicates P < 0.01. (**C**) DNA sequences of FHL124 (heterozygote T/C) and SRAO1/04 (homozygous C/C) human lens cell lines. (**D**) RNASeq quantitation of EPHA2 mRNA and Western blot showing EPHA2 protein levels in FHL124 and SRA01/04 cells. The Western blots shown were cropped before incubation with antibodies and full-length blots are not available.

The 1162 bp *EPHA2* promoter region was sequenced in 317 CTNS samples in which we had previously shown nearby *EPHA2-*related SNPs were associated with age related cataract^9^. A single SNP, rs6603883, was detected in this region in these individuals (Supplementary Fig. S1). While rs6603883 was not consistently associated with ARC in all populations (data not shown), because of its position it still seemed likely that it might influence transcription of *EPHA2*. To address this question, the *EPHA2* 1162 bp promoter region containing the TT or CC homozygous rs6603883 genotype was cloned into a luciferase reporter vector and transcriptional activity was measured by a dual-luciferase reporter assay 48 or 72 hours after transfection (Fig. 1A,B). As compared with the rs6603883 TT genotype, the transcriptional activity of *EPHA2* rs6603883 CC genotype was decreased about 33.5% and 36% at 48 hours or 72 hours after transfection respectively (P < 0.01). Thus, the rs6603883 CC genotype, decreases the transcriptional activity of the EPHA2 promoter. To confirm this observation EPHA2 mRNA and protein levels were measured in the FHL124 cell line, which is heterozygous (CT) for the rs6603883 genotype and SRA01/04 cell line, which is homozygous for the CC allele of rs6603883 (Fig. 1C,D). EPHA2 mRNA was approximately 2.3-fold higher in the FHL124 than SRA01/04 cells, and the protein level show a more dramatic difference, with EPHA2 being present in very low levels in the SRA01/04 cells.

### *EPHA2* is predicated to be a target gene of PAX2

**Figure 2:**
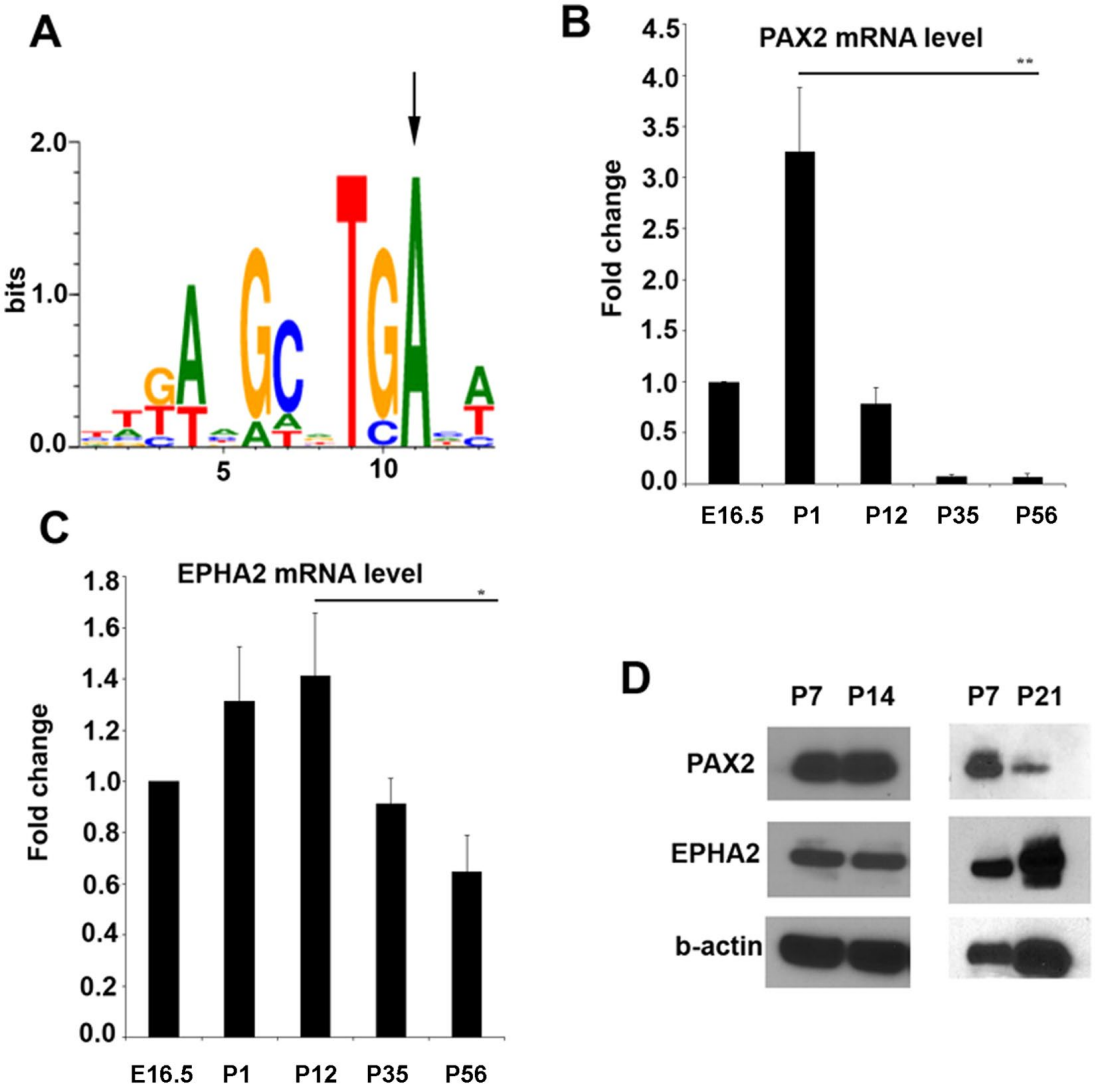
The EPHA2 promoter is predicted to contain a PAX2 binding site overlapping rs6603883 and both PAX2 and EPHA2 are developmentally expressed in lens. (**A**) Analysis using Genomatix predicated a PAX2 binding site in the *EPHA2* promoter. The T allelle of rs6603883, marked by the vertical arrow, is predicted to be 100% conserved in the PAX2 binding motif. (**B**,**C**) *Pax2* and *Epha2* mRNA levels in C57BL/6 mouse lenses were estimated by real-time PCR at different stages of development. (**D**) PAX2 and EPHA2 protein levels in the B6 mouse lens were detected by western blotting at P7, P14 and p21. Error bars represent standard deviation of 3 three independent experiments. * indicates P < 0.05, and ** indicates P < 0.01.

The molecular mechanism through which the rs6603883 C allele decreased transcriptional activity of the *EPHA2* promoter remained unclear. One was that it might affect binding of one or more transcription factors. To test this possibility, putative binding sites of transcription factors in the *EPHA2* promoter region were analyzed using the Genomatix program (https://www.genomatix.de). This analysis predicted the presence of a PAX2 (Paired box 2) binding site overlapping rs6603883, suggesting that PAX2 might be a means through which rs6603883 could directly affect the expression of *EPHA2* (Fig. 2A, marked with a vertical arrow). The rs6603883 T allele in the binding motif is predicted to be 100% conserved, suggesting the rs6603883 C allele might decrease PAX2 binding affinity, thus decreasing the transcriptional activity of *EPHA2*.

To address this possibility, the expression patterns of *Pax2* and *Epha2* in the C57BL/6 mouse lens were measured by real-time PCR of total RNA isolated at different developmental stages (Fig. 2B,C). While both *Pax2* and *Epha2* mRNA were present at E16.5, the level of *Pax2* mRNA peaked at P1, decreasing significantly by P12 and later stages. *Epha2* mRNA continued to increase through P12, decreasing at P35 and P56. To confirm their expression in lens, Pax2 and Epha2 protein levels were estimated by western blotting (Fig. 2D). Both Pax2 and Epha2 proteins can be detected in P7 and P14 lenses, confirming that the Pax2 and Epha2 proteins are expressed in the lens *in vivo*. Consistent with the real-time PCR results, the level of Pax2 protein has begun to decrease in the P21 mouse lens, while the level of Epha2 protein continued to increase through P21. Thus, the expression patterns of Pax2 and Epha2 in the mouse lens are consistent with the hypothesis that Pax2 might regulate Epha2 expression by inducing transcription of the Epha2 gene.

### PAX2 regulates EPHA2 mRNA and protein expression patterns of *PAX2* and *EPHA2* in the lens

**Figure 3:**
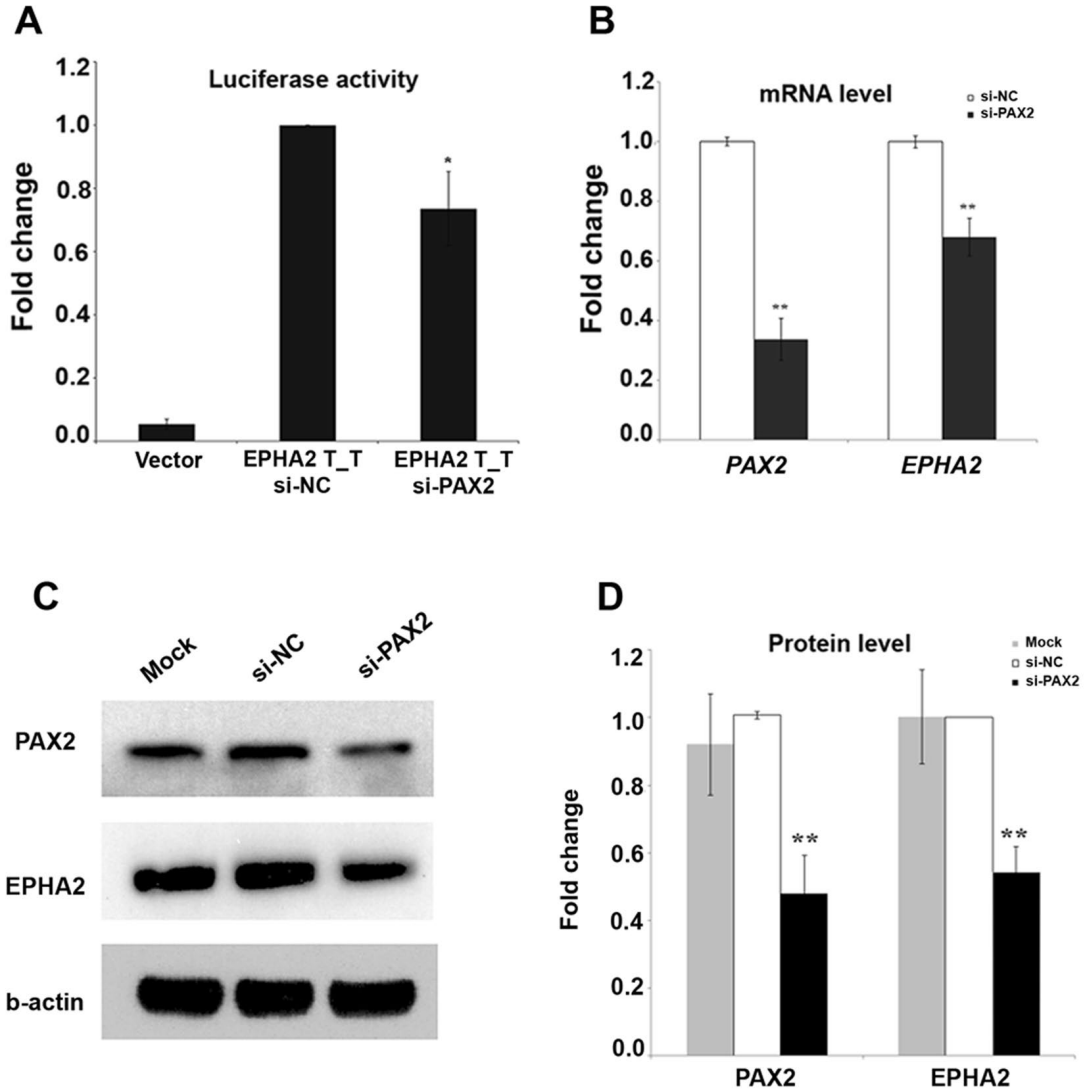
PAX2 regulates EPHA2 expression in HLE cells. (**A**) EPHA2 is reduced by transfection with si-PAX2: HLE cells were transfected with PGL4-EPHA2 pro. T_T and si-PAX2 and luciferase activity was carried out 48 hours later. (**B**) *EPHA2* mRNA levels decreased in *PAX2* knockdown HLE cells: mRNA level was tested by Real-time PCR 48 hours after transfection with si-NC or si-PAX2. (**C**) EPHA2 protein levels decreased in PAX2 knockdown cells. PAX2 and EPHA2 protein levels were estimated by Western blotting. (**D**) Statistical results of scanning and quantitating C. si-NC was a negative control used in si-RNA knockdown experiments. Error bars represent the standard deviation of 3 three independent experiments. * indicates P < 0.05, ** indicates P < 0.01.

The above data demonstrated *PAX2* is expressed in lens contemporaneously with *EPHA2* and the position of rs6603883 lies in a PAX2 binding site, but did not prove that PAX2 could regulate endogenous expression of *EPHA2* mRNA and protein in lens epithelial cells. To address this question, *PAX2* was knocked down in HLE cells by a specific siRNA (Fig. 3), after which EPHA2 levels were reduced by approximately 26.4% from control levels as estimated by a luciferase assay (Fig. 3A). *EPHA2* mRNA and protein levels were then measured 48 hours after siRNA transfection by real-time PCR and western blotting respectively. *PAX2 mRNA levels were* knockdown by about 66%, with an accompanying decrease in *EPHA2* mRNA level of about 32.3% (Fig. 3B). Protein levels also decreased significantly, to about half of control levels (Fig. 3C,D). These results demonstrated PAX2 not only regulates the transcriptional activity of the *EPHA2* promoter but also regulates of EPHA2 protein expression in HLE cells.

### rs6603883 alleles affect PAX2 regulation of *EPHA2* expression

**Figure 4:**
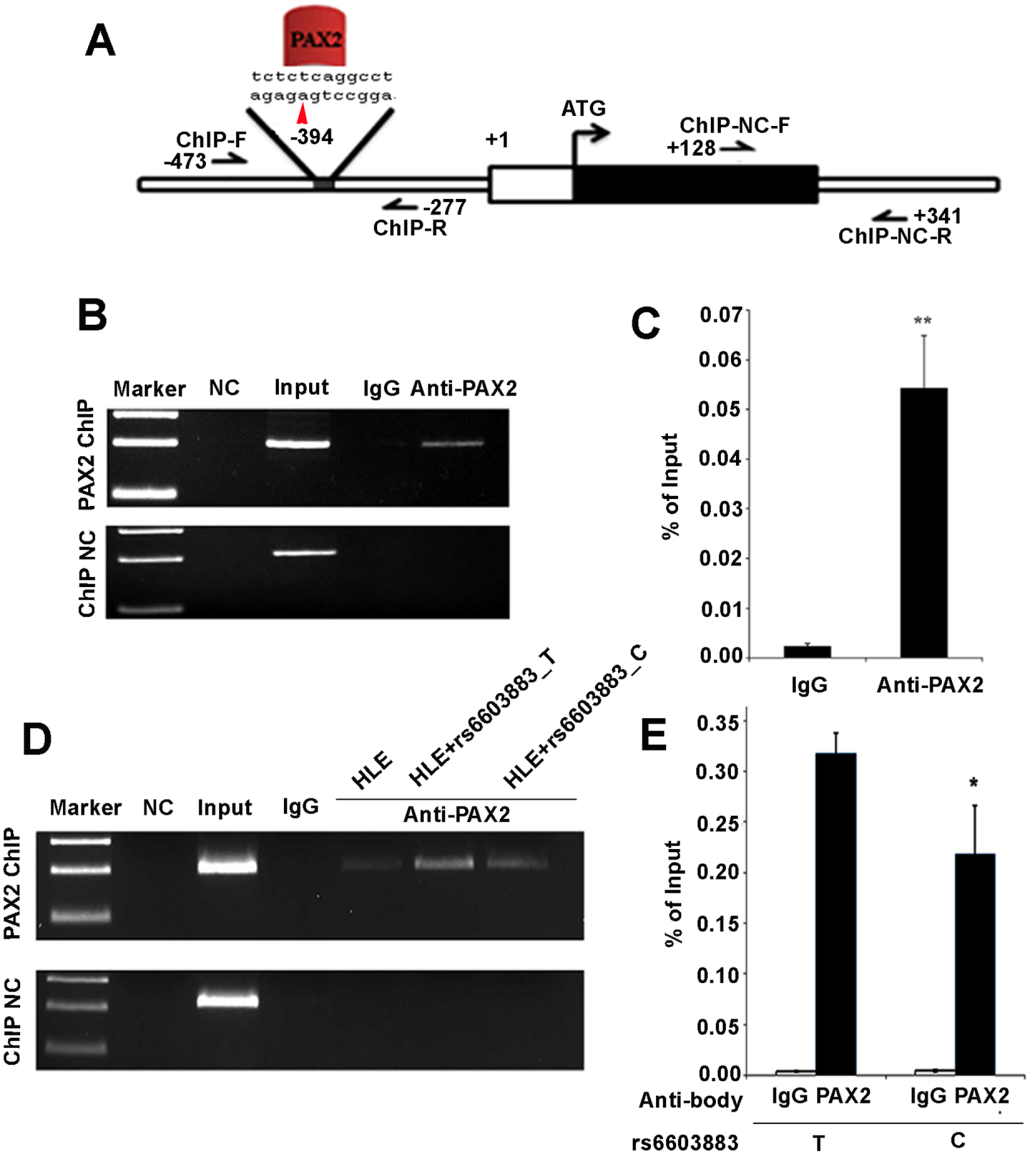
The rs6603883 C allele decreases the binding affinity of PAX2 to the *EPHA2* promoter. (**A**) A diagram of the *EPHA2* gene promoter showing the PAX2 binding site containing rs6603883 (red). ChIP-F and ChIP-R show the region for ChIP-PCR and ChIP-NC-F and ChIP-NC-R are primers used for the negative control. (**B**) ChIP-PCR analyzed anti-PAX2 (top) and ChIP-NC-PCR (bottom) pull down samples in HLE cells. Input is genomic DNA as positive control and IgG is the negative control for nonspecific binding. A specific PCR band can be seen in the anti-PAX2 pull down group samples. (**C**): PAX2 ChIP in HLE cells shows enrichment of the *EPHA2* promoter compared to IgG. (**D**) The PAX2 ChIP experiment was carried out in HLE cells transfected with an *EPHA2* promoter containing an rs6603883-T or rs6603883-C allele. (**E**) Compared with rs6603883-T, the rs6603883-C promoter has less enrichment by PAX2 ChIP. Error bars represent the standard deviation of 3 three independent experiments. * indicates P < 0.05, and ** indicates P < 0.01.

Given the expression patterns of *PAX2* and *EPHA2* in the lens, the effect of rs6603883 on EPHA2 expression, and the location of rs6603883 in a presumptive PAX2 binding site, it seemed likely that the C and T alleles of rs6603883 might have different binding affinities for PAX2. To test this, ChIP-PCR was used to analyze PAX2 binding to the *EPHA2* promoter region containing rs6603883 (Fig. 4A). A ChIP-PCR positive PCR band can be observed in the anti-PAX2 pull down group sample but not in the IgG control sample (Fig. 4B,C). When *EPHA2* promoter sequences containing the rs6603883 T allele or C allele were transfected into HLE cells and ChIP-PCR analysis was carried out 48 hours after transfection, enrichment of *EPHA2* containing the rs6603883 C allele decreased about 31.3% as compared to the T allele (Fig. 4D,E). This suggested that the C allele reduced the binding affinity of PAX2, providing a possible mechanism through which it decreases *EPHA2* transcription.

### RNA-seq Analysis of EPHA2 knockdown in HLE cells

**Figure 5:**
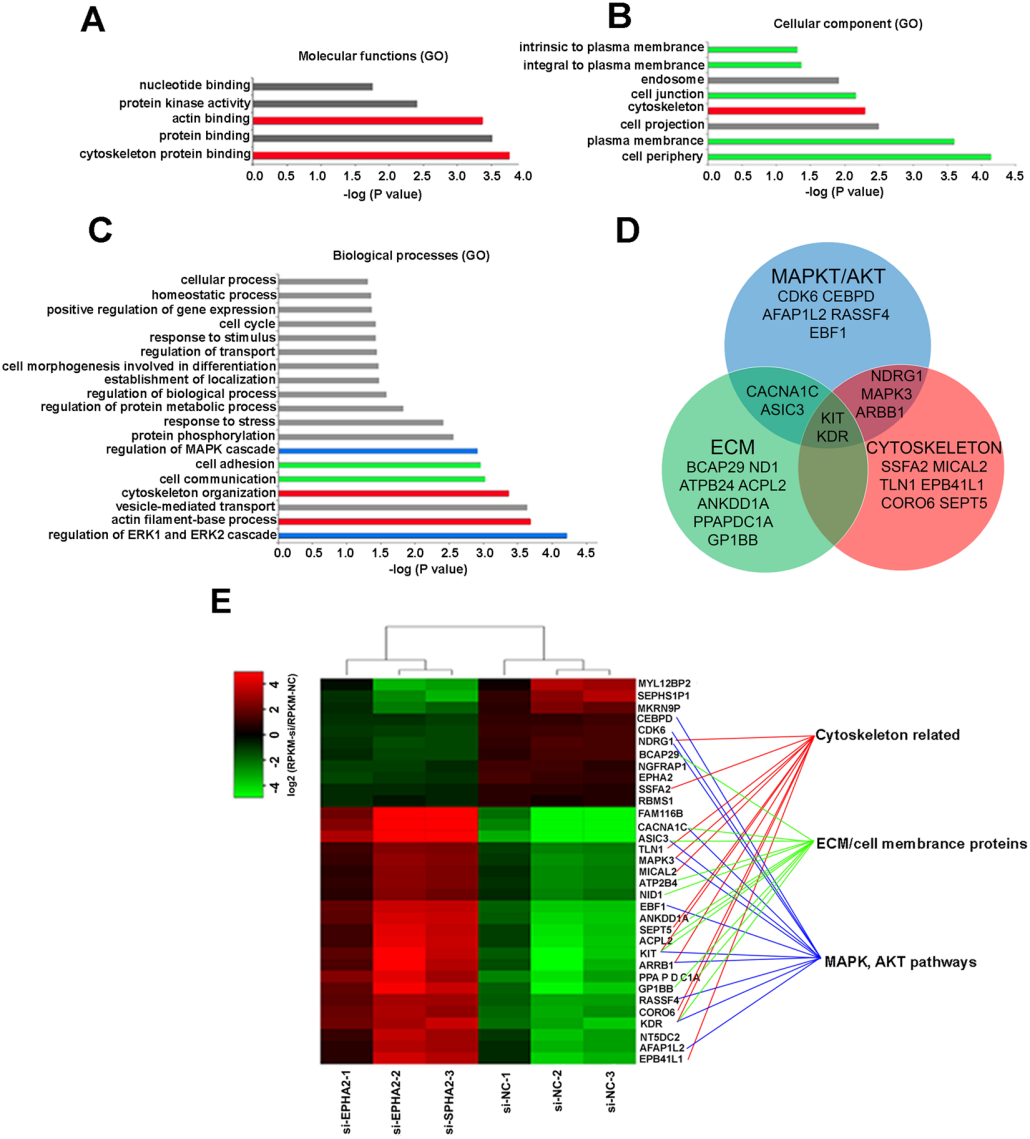
Knockdown of *EPHA2* affects the expression of ECM, cytoskeletal, and MAPK, AKT signaling pathway related genes. GO term enrichment analysis for RNA-seq genes for which the fold change is >2 and the adjusted p value is < 0.05. The analysis is based on (**A**) Molecular functions, (**B**) Cellular components and (**C**) Biological processes. (**D**) Venn diagram showing the distribution of differentially regulated genes among the ECM, cytoskeletal, and MAPK, AKT signaling pathway and the overlap among these groups. (**E**) RNA-seq Heat map of the gene expression profile form si-NC and si-EPHA2 treated HLE cells. Genes related to the ECM were marked with a green line, genes related to the cytoskeleton were marked using a red line, and genes related to the MAPK/AKT signaling pathway were marked using a blue line.

Although it is well established that *EPHA2* mutations or dysfunction will cause cataract, the functional roles of *EPHA2* in lens and the precise mechanisms through which *EPHA2* dysfunction induces cataract remain largely unknown. To elucidate possible downstream effects of *EPHA2* in HLE cells, *EPHA2* was knocked down using a specific siRNA and the resulting transcriptional changes were analyzed by whole transcriptome RNA sequencing (RNA-seq). Scanning of Western blots showed the level of EPHA2 protein was decreased approximately 80% (Supplementary Fig. S2). RNA-seq yielded an average of over 64 million paired end reads from each of three test and control samples, of which 78.6% were mapped to the human genome (Supplementary Table S1). Analysis of the RNA-seq data identified 33 genes that were differentially expressed (>2.0 fold, FDR p < 0.05) between si-control and si-*EPHA2* knockdown HLE cells: 11 genes were down regulated while 22 were up regulated (Fig. 5E, Table 1), which also showed that the expression level of *EPHA2* mRNA decreased significantly to approximately 33% of the control value (adjusted p < 1.2 × 10^−3^). Pathway analysis showed that many of the differentially expressed genes were active in the MAPK and AKT signaling pathway, were components of the extracellular matrix or plasma membrane, or were cytoskeletal proteins. In fact, this categorization is somewhat artificial, as there is significant overlap between these groups, with a number of the proteins belonging to two or even all three groups (Fig. 5D).

**Table 1:** RNA-seq results of transcripts showing significant changes (FDR p < 0.05, fold change ≥2) in siEPHA2 treated as compared to siNC treated HLE cells.

Symbol	p-value	FPKM adj. p-value	log2(fold change)*	mean RPKM (treated)	SD RPKM (treated)	mean RPKM (ctrl)	SD RPKM (ctrl)
ACPL2	1.91E-05	3.57E-02	2.05	3.46175	1.17992	0.45691	0.32194
AFAP1L2	3.32E-05	4.54E-02	1.71	3.40993	1.19869	0.62843	0.23474
ANKDD1A	8.32E-06	2.58E-02	2.06	0.78293	0.1184	0.10261	0.06711
ARRB1	2.83E-05	4.12E-02	2.07	2.78038	1.13803	0.35164	0.25685
ASIC3	1.61E-05	3.52E-02	2.58	0.87664	0.14663	0.02294	0.03244
ATP2B4	4.46E-06	2.05E-02	1.31	21.6024	2.29602	6.06637	2.85599
BCAP29	1.79E-05	3.57E-02	−1.64	3.05381	0.20926	6.89445	0.78496
CACNA1C	1.93E-05	3.57E-02	2.52	0.98926	0.24227	0.03964	0.05605
CDK6	3.30E-07	8.81E-03	−1.65	4.32572	0.45954	9.69674	0.86399
CEBPD	4.81E-06	2.05E-02	−1.5	5.06651	0.79479	10.23057	0.81812
CORO6	9.87E-06	2.87E-02	1.82	2.22179	0.45807	0.37716	0.19179
EBF1	1.08E-07	5.77E-03	2.1	7.51197	0.30971	1.09763	0.76938
EPB41L1	3.24E-05	4.46E-02	1.77	13.2809	3.57899	2.49485	1.73396
EPHA2	1.50E-08	1.20E-03	−1.58	10.40215	1.2575	22.33592	4.06042
FAM116B	1.48E-05	3.40E-02	2.55	0.91616	0.31208	0.04083	0.05774
GP1BB	1.03E-05	2.88E-02	2.44	1.54141	0.52104	0.10736	0.09937
KDR	2.43E-05	3.88E-02	1.94	0.24099	0.01652	0.0342	0.01857
KIT	9.41E-07	1.67E-02	2.33	2.48625	0.82701	0.25123	0.15155
LOC391722	5.90E-06	2.05E-02	−2.49	1.17436	0.17398	7.27198	3.94511
MAPK3	7.95E-07	1.67E-02	1.45	12.26905	1.66842	3.02164	1.24101
MICAL2	5.07E-06	2.05E-02	1.3	49.41406	4.68233	14.09715	6.92338
MKRN9P	2.49E-05	3.90E-02	−2.06	0.84955	0.10107	2.96485	1.08371
NDRG1	2.95E-05	4.20E-02	−1.63	2.22622	0.23821	5.0687	0.56539
NGFRAP1	5.61E-06	2.05E-02	−1.51	35.44579	3.70457	71.65926	2.91803
NID1	3.84E-05	4.99E-02	1.14	26.02729	1.59628	8.3188	4.00117
NT5DC2	3.67E-06	2.05E-02	1.88	18.50484	6.50047	2.9991	1.1559
PPAPDC1A	1.23E-08	1.20E-03	2.36	2.72354	0.52074	0.28686	0.08376
RASSF4	1.65E-06	1.94E-02	1.85	0.86469	0.1234	0.14519	0.05296
RBMS1	1.32E-05	3.40E-02	−1.12	7.30775	0.63744	11.14714	1.92924
SEPHS1P1	2.75E-05	4.07E-02	−2.36	0.26222	0.04069	1.59482	0.86369
SEPT	1.93E-05	3.57E-02	2.02	1.48439	0.48826	0.20058	0.12373
SSFA2	2.05E-05	3.57E-02	−1.22	8.49588	1.47447	14.12501	2.97129
TLN1	1.82E-06	1.94E-02	1.35	43.73144	1.92561	11.90739	5.83308

*log2 fold change of up-/down-regulated transcripts/loci.

### EPHA2 affects MAPK, AKT signaling pathways in HLE cells

Analysis of changes in biological processes in the differentially expressed gene list (Table 1) using Gene Ontology (GO) analysis showed enrichment of MAPK/ERK signaling pathway related genes (Fig. 5C, shown in blue), some members of which were also associated with the cytoskeleton (red) and extracellular matrix (green). As the AKT and MAPK signaling pathways undergo crosstalk to influence various cellular processes, the differentially expressed genes were included whether they were linked to either based on published data. Expression of 12 genes related to MAPK, AKT signaling pathways was significantly altered in *EPHA2* knock down HLE cells (Fig. 5D, blue lines, Table 2), Including *MAPK3*. MAPK and AKT signaling pathways have been shown to interact in playing critical roles in a variety of cellular processes including cell proliferation and cytoskeletal organization (Fig. 5A,B,C). In addition, *CEBPD* has been demonstrated to regulate the expression of α-tubulin directly^30^. These results suggested that decreased levels of *EPHA2*

**Table 2:** Detailed information and description of altered transcripts in specific pathways.

Gene	log2 (fold change)	P value	FDR	Function
**Transcripts involved in MAPK, AKT signaling pathway altered in** ***EPHA2*** **knockdown HLE cells**				
CDK6	−1.65	3.30E-07	8.81E-03	Mediates AKT signaling in cell cycle regulation
NDRG1*	−1.63	2.95E-05	4.20E-02	Inhibits the phosphorylation of AKT and ERK1/2^64^
CEBPD	−1.5	4.81E-06	2.05E-02	PI3-kinase/p38(MAPK)/CREB regulates the expression of CEBPD^65^
MAPK3*	1.45	7.95E-07	1.67E-02	Member of the MAP kinase family
AFAP1L2	1.71	3.32E-05	4.54E-02	Regulates AKT phosphorylation^66^
RASSF4	1.85	1.65E-06	1.94E-02	RASSF4 accelerates inhibition of the AKT phosphorylation by EV71^67^
KDR*^,†^	1.94	2.43E-05	3.88E-02	VEGF and KDR regulate AKT activity and MAPK pathway^68^
ARRB1*	2.07	2.83E-05	4.12E-02	Regulates Akt phosphorylation^69^
EBF1	2.1	1.08E-07	5.77E-03	Regulates the phosphorylation AKT and ERK^70^
KIT*^,†^	2.33	9.41E-07	1.67E-02	Regulates AKT and MAPK activity^71^
CACNA1C^†^	2.52	1.93E-05	3.57E-02	knockdown of CACNA1C in *Pkd1*−/− and *Pkd2−/−* cells altered Akt and Erk phosphorylation^72^
ASIC3^†^	2.58	1.61E-05	3.52E-02	p-AKT increased in *Asic3*−/− mouse^73^
*also member of cytoskeleton group, ^†^also member of the ECM group.				
**Extracellular matrix and cell surface related genes altered in EPHA2 knockdown HLE cells**				
BCAP29	−1.64	1.79E-05	3.57E-02	Integral component of plasma membrane (ENTREZ Gene: BCAP29)
NID1	1.14	3.84E-05	4.99E-02	Plays a role in cell interactions with the extracellular matrix (Entrez Gene: NID1)
ATP2B4	1.31	4.46E-06	2.05E-02	ATPase plasma membrane Ca2+ transporting 4, calcium transporter (Entrez Gene: ATP2B4)
KDR*^,†^	1.94	2.43E-05	3.88E-02	VEGF and KDR regulate AKT activity and MAPK pathway^70^
ACPL2	2.05	1.915-05	3.57E-02	Dephosphorylates xylose in the glycosaminoglycan protein linkage region of proteoglycans^76^
ANKDD1A	2.06	8.32E-06	2.58E-02	Ankyrin repeat and death domain containing 1A (Entrez Gene: ANKDD1A)
KIT*^,†^	2.33	9.41E-07	1.67E-02	Regulates AKT and MAPK activity^73^
PPAPDC1A	2.36	1.23E+08	1.22E-03	phospholipid phosphatase 4, Integral plasma membrane protein (Entrez Gene: PLPP4)
GP1BB	2.44	1.03E-05	2.88E-02	Glycoprotein Ib (platelet), beta polypeptide, NCBI ECM-receptor interaction
CACNA1C*	2.52	1.93E-05	3.57E-02	knockdown of CACNA1C in Pkd1−/− and Pkd2−/− cells altered Akt and Erk phosphorylation^74^
ASIC3*	2.58	1.61E-05	3.52E-02	p-AKT increased in Asic3−/− mouse^75^
*also member of the MAPK, AKT group, ^†^also member of the cytoskeleton group.				
**Transcripts involved in cytoskeleton organization altered in** ***EPHA2*** **knockdown HLE cells**				
NDRG1*	−1.63	2.95E-05	4.20E-02	Inhibits actin-filament polymerization, stress fiber assembly and formation^74^
SSFA2	−1.22	2.05E-05	e.57E-02	Actin binding membrane protein^75^
MICAL2	1.3	5.07E-06	2.05E-02	Inhibits actin stress fibers and actin microfilament^76^
TLN1	1.35	1.82E-06	1.94E-02	Required for stress-fiber formation, as well as microtubule assembly^77^
MAPK3*	1.45	7.95E-07	1.67E-02	Many ERK1/2 molecules are tethered to cytoskeletal elements such as microtubules and actin filaments^78^
EPB41L1	1.77	3.24E-05	4.46E-02	Mediates interactions between the erythrocyte cytoskeleton and the overlying plasma membrane^79^
CORO6	1.82	9.87E-06	2.87E-02	Coronin is an actin binding protein, interact with microtubules^80^
KDR*	1.94	2.43E-05	3.88E-02	VEGF through KDR increases polymerized F-actin fibers^81^
SEPT5	2.02	1.93E-05	3.57E-02	Regulates cytoskeletal organization (Entrez Gene: SEPT5 septin 5)
ARRB1*	2.07	2.83E-05	4.12E-02	Knockdown of *ARRB1* reduces RhoA activation and stress-fiber formation^82^
KIT*	2.33	9.41E-07	1.67E-02	Regulates the actin cytoskeleton and promote filopodia formation through WASP^83^
*also member of the MAPK, AKT group, ^†^also member of the ECM group.				

might induce cataract by causing changes in the MAPK and AKT signaling pathways with resultant dysfunction pathways they regulate in lens epithelial cells.

### EPHA2 affects expression of ECM and cell surface related genes

As the ECM has been demonstrated to be active in MAPK and AKT signaling pathways through cell membrane receptors and channels^31^, it seemed possible that when EPHA2 is knocked down changes in expression of ECM and cell surface components might be associated with alterations of the MAPK/AKT-pathways. GO analysis of both cellular components and biological processes confirmed this (Fig. 5B and C). Of the 33 genes whose expression was significantly altered by knockdown of EPHA2, 11 of them were related to the ECM or cell surface, not including EPHA2 itself (Fig. 5D, green lines, Table 2). Some of these were also active in MAPK and AKT signaling or related to the cytoskeleton. These include two receptors (*c-KIT* and *KDR* (also named *VEGFR*)); three cell membrane channel related proteins *ASIC3*, *ATP2B4*, and *CACNA1C*; and 4 ECM related genes (*NID1*, *ACPL2*, *ANKDD1A*, and *GP1BB*). In addition, this group included *BCAP29*, a membrane chaperone active in processing and trafficking P-glycoprotein 1 (permeability glycoprotein, Pgp) to the cell surface; and *PPAPDC1A*, a plasma membrane phospholipid phosphatase; and *KDR*, which is also active in the MAPK, AKT pathways.

### EPHA2 affects expression of cytoskeleton related genes

Cytoskeleton related genes were enriched based on molecular functions, cellular component, and biological processes analysis (Fig. 5A–C). Including both the GO analysis results and published papers, a total of 11 genes whose expression changed significantly in *EPHA2* knockdown HLE cells were related to cytoskeleton organization or regulation (Fig. 5D, red lines, Table 2). Most of these proteins interact with actin filaments and stress fibers, microtubules, or both. In addition, EPB41L1 mediates interactions between the cytoskeleton and plasma membrane, and SSFA2 is a filamentous actin-interacting protein required for localization and function of IP(3)R to the endoplasmic reticulum, MAPK3 is an ERK molecule tethered to actin filaments, and ARRB1 is involved in stress fiber formation. As the cytoskeleton plays an important role in lens development and transparency^32^, these results suggest EPHA2 might also exert effects through regulating the expression of cytoskeletal genes in HLE cells, affecting cytoskeleton organization and cellular shape and organization to contribute to cataract. Finally, transcripts for 3 presumptive pseudogenes and 4 additional genes, two of which are involved in cell death signaling, were altered in EPHA2 knockdown HLE cells (Table 1, Supplementary Table S2).

## Discussion

While genetic influences on ARC are well documented^33^, the specific genes and mechanisms of these effects are only beginning to be elucidated. Polymorphisms in the *EPHA2* region have been shown to be associated with ARC^9, 12, 13, 16^, but the mechanisms through which these polymorphisms and *EPHA2* itself affect ARC are still largely unknown. Having identified no changes in the *EPHA2* coding sequence in ARC patients in the CTNS, it seemed reasonable to examine the promoter sequences, which might be expected to contribute to ARC through regulating the gene’s transcriptional activity. Sequencing of 1162 bp of the *EPHA2* promoter in the CTNS samples identified a single SNP, rs6603883. Additional SNPs exist in the 1162 bp promoter region, but have overall allele frequencies well below 1% in Europeans (http://www.1000genomes.org/1000-genomes-browsers and were thus not felt likely to contribute significantly to differences in *EPHA2* expression in this population overall. rs6603883 Lies in a PAX2 recognition motif, and the C allele decreased the binding affinity of PAX2 and thus decreased the transcription of *EPHA2*. Measurement of EPHA2 mRNA and protein in human lens cell lines also suggested that the CC rs6603883 allele decreases levels of both EPHA2 mRNA and protein, although these cell lines show a number of differences in gene and protein expression, so that the rs6603883 allele might be only one of many factors affecting these levels. This is particularly true of the protein levels, which are disproportionately lower in the SRA01/04 cells relative to the mRNA levels. Knockdown of *PAX2* in HLE cells decreased expression of *EPHA2*, suggesting *EPHA2* is one of the PAX2’s target genes. Finally, knockdown of EPHA2 in HLE cells affected expression of genes in the MAPK/AKT regulatory pathways and thence genes in the ECM and cytoskeleton groups, suggesting involvement of these pathways possible rs6603883 influences on ARC.

As metazoans evolved ocular and nervous systems, the ancestral single *PAX* gene diverged into *PAX6*, *PAX6(5a)*, and *PAX2*. While *PAX2* is highly expressed and well-studied in the optic nerve, its functions in the lens are subtler and remain poorly understood. Although *Pax2* cannot replace *Pax6* in lens induction, lenses of *Pax6+/−* mice are normal in size, while *Pax2*−/−; *Pax6* /− mouse lenses are rudimentary^19, 34–36^, Implicating PAX2 in lens development. *PAX2* also regulates expression of the crystallin protein in the Drosophila lens^23^. Consistent with this, our data demonstrated PAX2 is expressed in the mouse lens and regulates the expression of *EPHA2*. Developmentally, *Pax2* began to decrease in the mouse lens by P12, while *Epha2* was still highly expressed until decreasing at P60 (Fig. 2B,C), suggesting that other transcription factors in addition to *PAX2* might help regulate *EPHA2* expression in the lens. In this regard, transcription factors HOXA1 (homeobox A1), HOXB1 (homeobox B1), P53 (tumor protein p53) and HIC1 (hypermethylated in cancer 1) have been reported to regulate the transcription of *EPHA2* directly^37–40^. P53 is known to regulate c-Maf, Prox-1, *CRYAA*, and *CRYBA3* expression during lens development and helps regulate apoptosis and progression of the cell cycle^41, 42^, but whether the other factors are active in the lens remains to be demonstrated.

**Figure 6:**
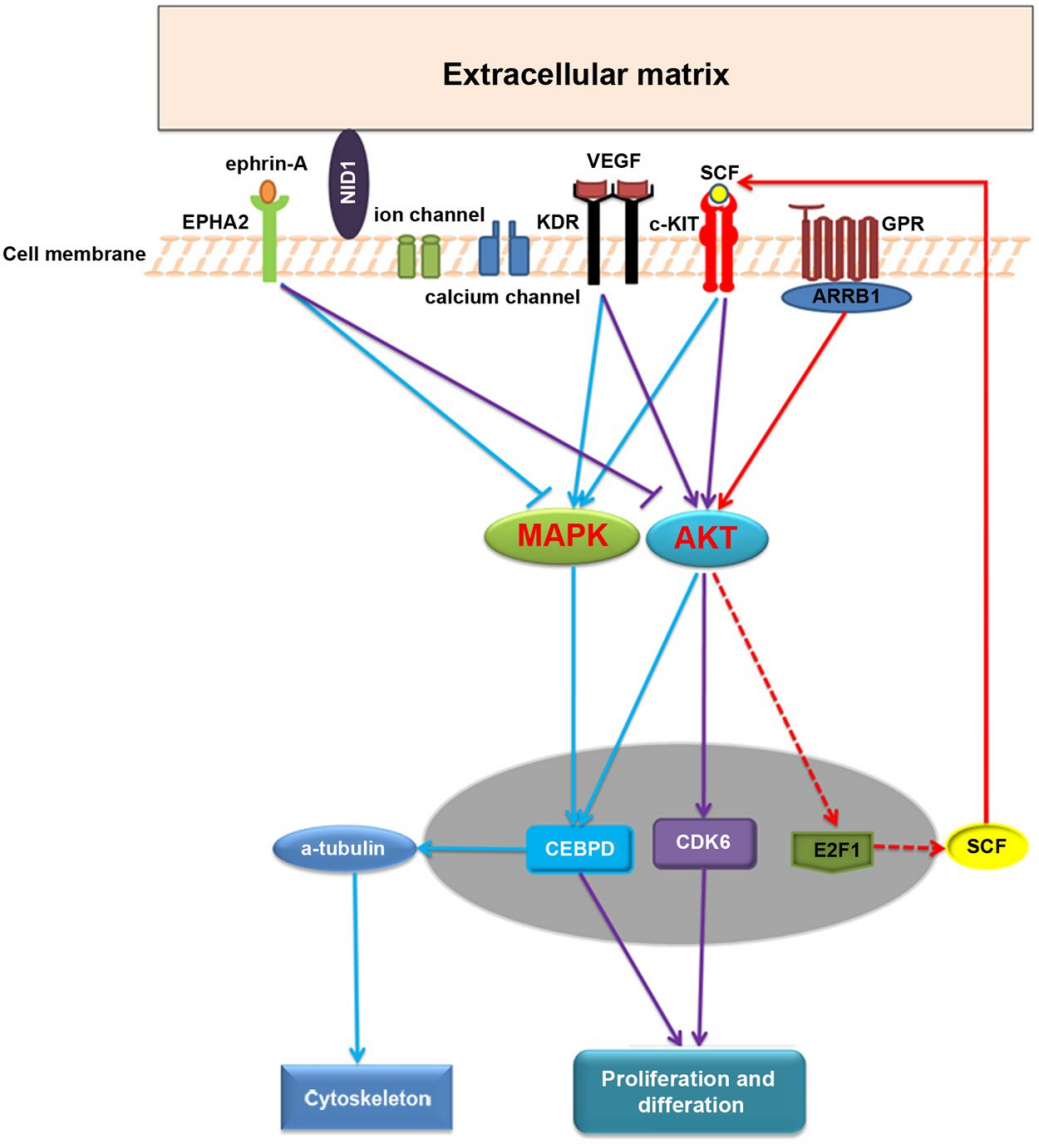
Diagram showing the MAPK and AKT signaling pathways changed in *EPHA2* knockdown HLE cells. Components displayed were differentially expressed in the RNA-seq analysis with >2 fold changes and adjusted p values < 0.05 except E2F1 and SCF, which are marked with a dotted line.

EPHA2 previously has been reported to regulate the MAPK and AKT signaling pathways^16, 43, 44^. These pathways have been demonstrated to be related to cell differentiation, proliferation, migration, and anti-oxidant activity in the lens. Erk activation is required for lens fiber differentiation^45^. They also have been implicated in cataractogenesis. AKT was highly elevated in *PTEN* knockout lenses that have cataract^46^, and mice expressing constitutively active Mek1, an activator of Erk1 and Erk 2 kinases, show cataract and macrophthalmia, probably through elevated glucose transport and levels^47^, as both MAPK and AKT signaling pathways were increased in osmotic stress induced sugar cataract^48^. Consistent with these results, our RNA-seq result revealed that knockdown of *EPHA2* in HLE cells induced differentially expressed genes that are part of the MAPK and/or AKT signaling pathways (Fig. 5C,D; Table 2). This result suggested EPHA2 may act through effects on the MAPK, AKT signaling pathways to cause HLE cell dysfunction and finally to induce cataract (Fig. 6).

Both the MAPK and AKT signaling pathways can be regulated by the ECM through specific receptors or cell membrane channels^31^. In addition, the extracellular matrix (ECM) plays an important role in lens structure and function, and mutations in ECM genes have been shown to be associated with cataract^49, 50^. Consistent with this result, in addition to the MAPK and AKT signaling pathways, knocking down EPHA2 levels resulted in significant changes in the expression of 11 genes related to the ECM, cell membrane, cell surface, or basement membrane. These included four ECM related genes, two cell membrane receptors, three membrane channels, as well as BCAP29, a chaperone influencing processing and trafficking of Pgp to the cell surface, and PPAPDC1A (PLPP4), an integral membrane phospholipid phosphatase active in signal transduction. Among the 4 ECM related genes, an *NID1* mutation in *Romagnola Cattle* caused inherited cataracts^51^, and the VEGF-KDR signaling pathway also has been reported to play important roles in cataractogenesis^52^. These results are consistent with the hypothesis that EPHA2 may act through the EMC and cell membrane to alter the MAPK and AKT signaling pathways, affecting the cytoskeleton and increasing susceptibility of the aging lens to cataract.

Eph receptors are known to play a role in remodeling the actin cytoskeleton through the Rho family of guanosine triphosphate hydrolases (GTPases)^53^. Activation of EPHA2 by ephrin-A1 can change the cytoskeletal morphology and cellular morphogenesis by controlling disassembly of the cytoskeleton^54, 55^. Our RNA-seq data also showed dysregulation of cytoskeletal genes in *EPHA2* knockdown HLE cells. One possibility is that knockdown of *EPHA2* in HLE cells affected the cytoskeleton organization by altering regulation of MAPK and AKT signaling pathways.

The MAPK and AKT signaling pathways play critical roles in a variety of cellular events, including cytoskeleton organization. Lens transparency depends on the organization of cytoplasmic, cytoskeletal and membrane proteins and cell-cell interactions. Cytoskeletal elements including microfilaments, microtubules and intermediate filaments are believed to play essential role in lens transparency^32^. Both actin and tubulin have been reported to be decreased in cataractous lenses^56^, and mutations in BFSP1 and BFSP2 have been reported to be associated with cataract in humans^57, 58^. Actin and actin-interacting proteins conceivably play vital roles in lens fiber cell elongation and differentiation, as disruption of the actin cytoskeleton has been reported to impair lens epithelial elongation and differentiation, resulting in alteration of lens cell shapes^32, 59^. In addition, CRYAA and CRYAB, in which mutations can cause cataract, can bind actin^60, 61^. It is also interesting that expression of three pseudogenes decreased in our RNA-Seq analysis (Supplementary Table S2). It seems possible that these might not actually be pseudogenes, but also participate in the MPAK, AKT signaling pathways or other pathways in which EPHA2 may act.

In summary, rs6603883 in the promoter region of *EPHA2* lies in the binding motif of PAX2 (paired box 2), and the C allele decreases binding of PAX2 to the *EPHA2* promoter with a resulting reduction in *EPHA2* transcription. In addition, knockdown of *PAX2* in HLE cells decreases levels of both *EPAH2* mRNA and protein. RNA sequencing showed that 33 genes were differentially expressed with a greater than a 2-fold change and an adjusted P value less than 0.05. Among these genes, 10 were related to cytoskeleton organization, 12 were related to the MAPK and/or AKT signaling pathways and 4 were ECM related genes. These results suggest that EPHA2 may act in HLE cells through ECM regulation of MAPK and AKT signaling pathways to affect cell cytoskeletal organization and induce cataract formation. Even though our current data do not elucidate the exact mechanisms of *EPHA2* in ARC susceptibility, they do suggest a regulatory axis of EPHA2-ECM-MAPK/AKT-cytoskeleton- cataract exists in HLE cells. Future studies will center on elucidation of the functional role of EPHA2 in the lens and cataract. These results will help us to understand the mechanisms of age related cataract, which potentially will allow development of potential methods to delay or even prevent ARC.

## Methods

### DNA samples

Genomic DNA was isolated from human blood samples using a standardized protocol that included cell lysis with anionic detergent, high salt precipitation of proteins, ethanol precipitation to concentrate DNA followed by further purification of DNA with a buffered phenol/chloroform mixture. After a final precipitation with alcohol the DNA pellet was dissolved in Tris-EDTA 10 mM, pH 8.0^62^. The tenets of the Declaration of Helsinki were followed. Informed consent was obtained, and the protocols for human experimentation were reviewed and approved by the Institutional Review Boards of the National Eye Institute and the Institute of Ophthalmology at the University of Parma.

### PCR and sequencing

1162 bp of the *EPHA2* promoter region were amplified using primers: EPHA2-promoter-F: CCGCTTCCCAAGAGTAGGCACCA; EPHA2-promoter-R: CCCTCCTGCCCCGAGTCCTTAAT. PCR reagents included: 10x PCR buffer: 1.0 ul, Mg^2+^ 0.6 ul, dNTP 0.5 ul, 10 pm primer 0.5 + 0.5 ul. Taq 1 u, DNA 40 ng, H_2_O up to 10 ul. Cycling including a touchdown PCR reaction for the first 15 cycles: 94 °C for 4 min, followed by decreasing the annealing temperature from an initial 64 °C in a stepwise fashion by 0.5 °C every second cycle, and 72 °C for 1.5 min. For the later 20 cycles: 94 °C for 40 sec, 57 °C for 30 sec and 72 °C for 1.5 min and finally a prolonged elongation step at 72 °C for 10 min. PCR production was purified and analyzed by Sanger sequencing using an ABI 3130 sequencer with Big Dye Terminator Ready reaction mix according to the manufacturer’s instructions (Applied Biosystems, Foster City, CA). Sequencing results were analyzed using Mutation Surveyor v3.30 (Soft Genetics, State College, PA) or Lasergene 8.0 (DNASTAR, Madison, WI).

### Cell culture and siRNA transfection

The HLE cell line (FHL124), which has 95% similarity in transcriptional profile to human lens epithelia^63^, was kindly provided by Dr. JR Reddan (Oakland University) and cultured in 1 g/L glucose DMEM contains 10% FBS. si-PAX2 (Sense: GGUCUUUCCAAGGUUGGGATT) or si-EPHA2 (Sense: GGUGCACGAAUUCCAGACGTT) were purchased from Invitrogen (Grand Island, NY). siRNA transfection was carried out by PepMute (SignaGen, Gaithersburg, MD) reagent as the follow: Cells were sub-cultured 1 day before transfection, the cell density reached to about 80%. 1 hour before transfection, cells were cultured in the fresh complete medium. For experiments in 6-well plates: 50 nM siRNA was mixed with 4 ul PepMute reagent into 100 ul transfection buffer. The mixed reagents were kept the at room temperature for about 15 min, and the transfection mixture was added to the cells and they were cultured for about 5 hours, after which the transfection culture medium was replaced with fresh complete culture medium. 48 hours later, knockdown efficiency or functional tests were carried out respectively.

### Luciferase Reporter Vector construction, plasmid transient transfection and Luciferase Reporter assays

The human *EPHA2* proximal promoter region was amplified by the primers EPHA2-Promoter-F and EPHA2-Promoter-R (above). The PCR product was then cloned into the PCR 2.1-TOPO (Invitrogen, Grand Island, NY) vector. After sequencing for verification, the *EPHA2* promoter region was then cloned into the PGL4.17 vector (Promega, Madison, WI) with the restriction enzymes of *Hind* III and *Xho*I (NEB, Ipswich, MA). HLE cells were grown to 80% confluence in 6-well plates. One ug of either PGL4-EPHA2 Promoter or PGL4 plasmid were transfected into HLE cells along with 30 ng pGL4.75 [hRluc/CMV] using a LipoJet Transfection Kit (SignaGen, Gaithersburg, MD). Forty-eight or Seventy-two hours after transfection luciferase activities were tested using dual-luciferase reporter assay system (Promega, Madison, WI) per the manufacturer’s suggested protocol.

### RNA isolation and real-time PCR

Mouse lens or HLE cell total RNA was isolated using Trizol (Life Technologies) and was reverse transcribed into cDNA using a reverse transcriptase kit (Invitrogen, Grand Island, NY) with random primers, and processed for real-time PCR using SYBR Green (Life technologies). Reactions were run in triplicate and data was normalized with *GAPDH*. Primers using for real-time PCR as: Human *GAPDH* F: AGGGCTGCTTTTAACTCTGGT; R: GACAAGCTTCCCGTTCTCAG. Human *PAX2* F: TGTGACTGGTCGTGACATGG, R: GGGAACTTAGTAAGGCGGGG. Human *EPHA2* F: GATCGGACCGAGAGCGAGAA; R: GGTCCCACCCTTTGCCATAC. Mouse *Gapdh* F: CGTCCCGTAGACAAAATGGT; R: TCAATGAAGGGGTCGTTGAT. Mouse *Pax2* F: CGAGTCTTTGAGCGTCCTTCCTA; R: GCAGATAGACTGGACTTGACTTC. Mouse *Epha2* CAAAGTGCACGAGTTCCAGA, R: CTCCTGCCAGTACCAGAAGC. All procedures with mice in this study were performed in compliance with the tenets of the National Institutes of Health Guideline on the Care and Use Animals in Research and the ARVO Statement for the Use of Animals in Ophthalmic and Vision Research.

### Western blotting

HLE cells were washed with PBS and lysed on ice for 30 minutes with RIPA (Santa Cruz Biotechnology, Dallas TX). 20ug Total Protein was separated by SDS-PAGE and transferred onto PVDF membranes, blocked with 5% non-fat milk at room temperature for 1 hour, and incubated at 4 °C for overnight with either anti-EPHA2 (1:1000, Cell signaling), anti-PAX2 (1:600, Abcam, Cambridge, MA) or anti-beta-actin (1:4000, Abcam, Cambridge, MA). The primary antibodies were identified with the appropriate secondary antibody at room temperature for 2 hours. Quantification of protein bands was performed using ImageJ software (http://rsb.info.nih.gov/ij/index.html) and normalized to beta-actin.

### Chromatin immunoprecipitation

ChIP analysis was carried out with HLE cells or HLE cells transfected with plasmid containing the *EPHA2* promoter 48 hours later using ChIP-IT express Enzymatic Magnetic Chromatin Immunoprecipitation kit as the standard protocol (Active motif, Carlsbad CA). Antibodies used for ChIP include: Anti-Human IgG ChIP grade (Abcam, Cambridge, MA); Anti-PAX2 antibody ChIP grade (Abcam, Cambridge, MA). Primers used for ChIP PCR are: CHIP-F: TTTTGACCATCAGCAGCTTG; CHIP-R: CTGCCCTTCACCTCTGAGAC; and ChiP-NC F: GATCGGACCGAGAGCGAGAA, R: CGACACCAGGTAGGTTCCAA. Real-time PCR was used to test PAX2 ChIP enrichment.

### RNA sequencing

RNA from three biologically repeated si-NC and three si-EPHA2 transfected HLE cell experiments was isolated using Trizol (Invitrogen). Transcriptome expression profiling was analyzed by RNA sequencing using HiSeq™ 2000 platform (Illumina) by Beijing Genomics Institute (BGI, Hong Kong, China). The raw reads were analyzed by trimming filtering and the sequences were aligned to the human genome (hg19) using Genomatix mining station. Differentially expressed genes were identified by Genomatix (https://www.genomatix.de, USA: Ann Arbor, MI). Transcripts displaying >2.0 fold change and FDR (False discovery rate) adjusted P values < 0.05 were considered to be significantly differentially expressed.

### Statistical analysis

SNP genotype frequencies, Chi square p values, odds ratios with 95% confidence intervals, haplotype probabilities (by the CHM method), and HWE (Hardy–Weinberg equilibrium) were analyzed using the SVS software package (Golden Helix, Bozeman, MT). Since the SNP haplotype extended over only 334 bp recombination was assumed to be 0 for these markers. The odds ratios (OR) and 95% confidence intervals (CI) were calculated to estimate the strength of the association. The experiments of mRNA, protein and luciferase activity test were repeated three time and results were presented as mean ± standard deviation (SD). Statistical significance between experimental and control groups was assessed with Student’s t-test. P < 0.05 was considered significant. Gene Ontology (GO) analysis of the differentially expressed genes was carried out using Genomatix software (Ann Arbor, MI).

## Electronic supplementary material

Supplementary Information
