# Supplementary material for: Functional non-coding polymorphism in an EPHA2 promoter PAX2 binding site modifies expression and alters the MAPK and AKT pathways
Source: Sci Rep. 2017 Aug 30;7:9992. doi: 10.1038/s41598-017-10117-3 (PMC5577203; doi:10.1038/s41598-017-10117-3)
Supplement: Supplementary file 1 — Supplementary Information [file 41598_2017_10117_MOESM1_ESM.pdf]

Supplemental Information for:

Functional non-coding polymorphism in an *EPHA2* promoter PAX2 binding site modifies expression and alters the MAPK and AKT pathways

Xiaoyin Ma,[1, 2] Zhiwei Ma,[2] Xiaodong Jiao,[2] and J. Fielding Hejtmancik [2] *

[1]Laboratory of Developmental Cell Biology and Disease, School of Ophthalmology and Optometry and Eye Hospital, Wenzhou Medical University, 325003, China

[2]Ophthalmic Genetics and Visual Function Branch, National Eye Institute, National Institutes of Health, Bethesda, MD 20892, USA

*Correspondence to: J. Fielding Hejtmancik, MOGS/OGVFB/NEI/NIH, 5635 Fisher's Lane, Room 1127, Rockville, MD, 20852; Phone: (301) 496-8300; FAX: (301) 435-1598; email: f3h@helix.nih.gov

Supplementary Figure S1.

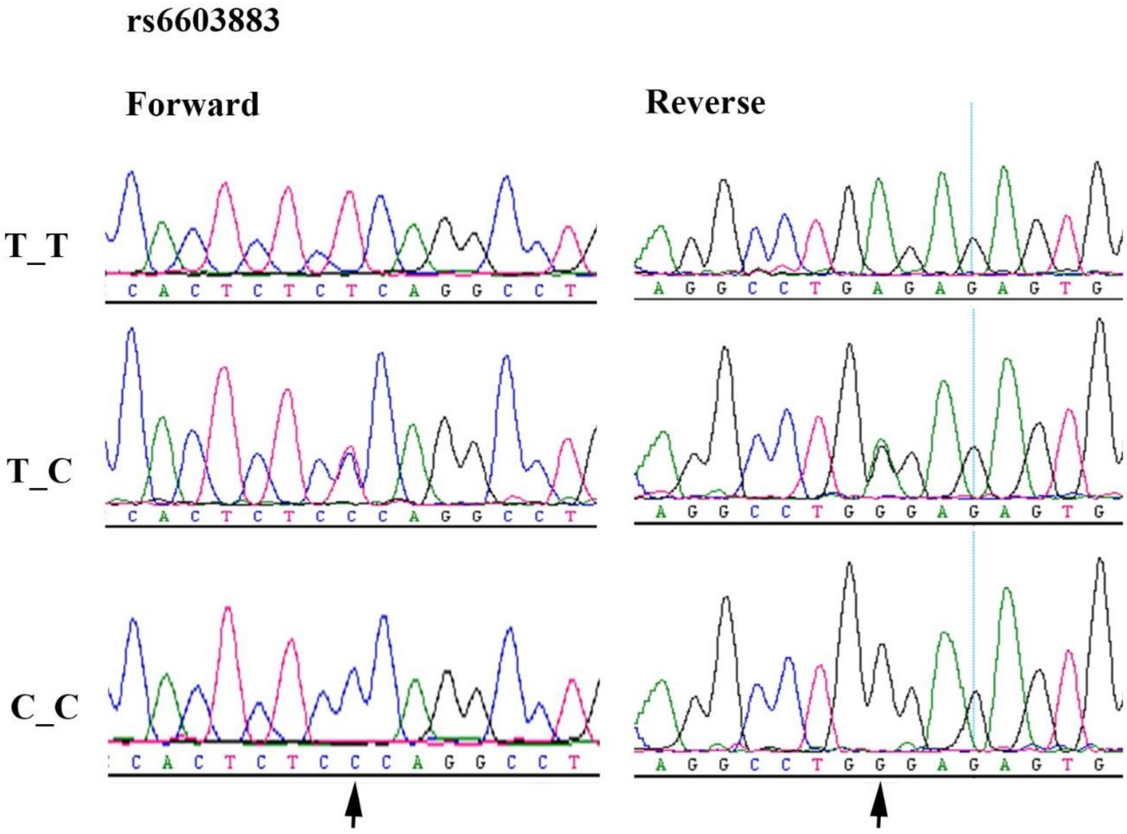

Supplementary Figure S1. Sequence tracings from individuals with various rs6603883 genotypes.

Supplementary Figure S2.

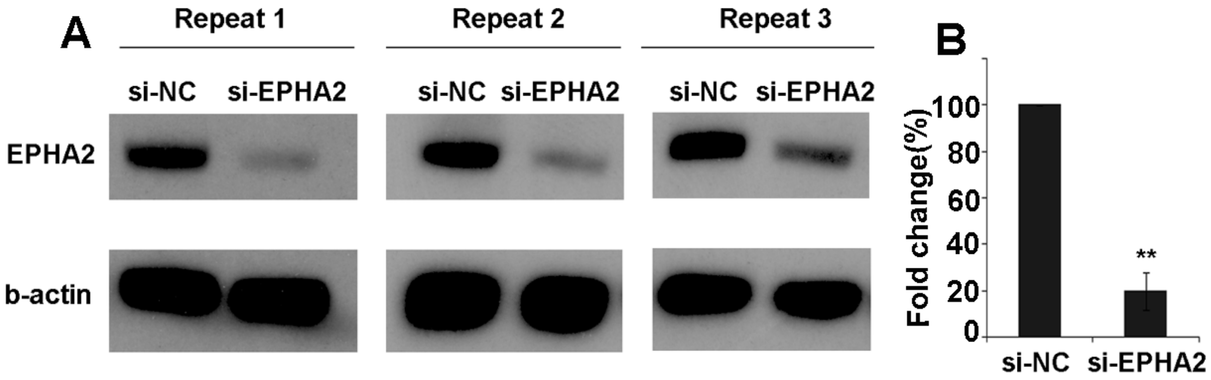

Supplementary Figure S2. Western blot shows efficiency of EPHA2 knockdown in HLE cells. EPHA2 protein level decreased significantly in si-EPHA2 treated cells compared with the si-NC. B: Quantitation of the western blot results shown in Fig. 6A based on analysis using image J software (the protein levels in si-NC treated cells were normalized as 100%).

Supplementary Table S1. Characteristics of RNA-seq analysis of EPHA2 knockdown cells.

Sample	total reads	average read length	Unique hits	percent of Unique hits
**si-EPHA2-1**	64,334,653	108.2	52,181,837	0.81
**si-EPHA2-2**	64,261,390	108.6	52,000,317	0.81
**si-EPHA2-3**	63,130,093	108.3	53,502,754	0.85
**si-NC-1**	64,515,829	108.5	50,935,247	0.79
**si-NC-2**	64,499,159	125	46,736,091	0.72
**si-NC-3**	64,247,461	125	47,080,540	0.73
**Averages**	64,164,764	113.9	50,406,131	0.79

Supplementary Table S2. Pseudogene transcripts altered in EPHA2 knockdown of HLE cells.

Genes	log2(fold change)	P value	FDR	Official Name
LOC391722	-2.49	5.90E-06	2.05E-02	myosin, light chain 12B pseudogene 2 (MYL12BP2)
SEPHS1P1	-2.36	2.75E-05	4.07E-02	selenophosphate synthetase 1 pseudogene 1
MKRN9P	-2.06	2.49E-05	3.90E-02	makorin ring finger protein 9, pseudogene
